# Exploring the immunological role and prognostic potential of *PPM1M* in pan-cancer

**DOI:** 10.1097/MD.0000000000032758

**Published:** 2023-03-24

**Authors:** Rongruo Zeng, Lulu Wang, Yuxu Zhang, Ye Yang, Jie Yang, Yan Qin

**Affiliations:** a Department of Health Management, The People’s Hospital of Guangxi Zhuang Autonomous Region & Research Center of Health Management, Guangxi Academy of Medical Sciences, Nanning, Guangxi, People’s Republic of China; b Department of Pathology, Guangxi Medical University Cancer Hospital, Nanning, Guangxi, People’s Republic of China; c Department of International Medicine Services, The People’s Hospital of Guangxi Zhuang Autonomous Region, Nanning, Guangxi, People’s Republic of China; d Department of Rehabilitation Medicine, Guangxi Medical University, Nanning, Guangxi, People’s Republic of China.

**Keywords:** human tumors, immune infiltration, immunotherapy, pan-cancer analysis, *PPM1M*, prognostic value

## Abstract

*PPM1M* is a member of the metal-dependent protein phosphatase family, and its role in the immunization process has not been studied in depth. In this study, we investigated the role of *PPM1M* in pan-cancer.

**Methods:**

Samples of cancer and normal tissues were obtained from the cancer genome atlas and genotype-tissue expression. Kaplan–Meier survival curves and Cox regression were used to analyze the effect of *PPM1M* on prognosis. Functional and pathway enrichment analyses were performed using the R package “clusterProfiler” to explore the role of *PPM1M*. The Sanger Box database was used to analyze the relationship between *PPM1M* and tumor immune checkpoint, tumor mutational burden, and microsatellite instability. The Tumor Immune Estimation Resource 2 database and CIBERSORT method were used to analyze the relationship between *PPM1M* and tumor-infiltrating immune cells. Finally, the cBioPortal database was used to analyze the genomic variation in *PPM1M.*

**Results:**

Among the variety of tumors, the expression of *PPM1M* was higher in normal tissues than in cancerous tissues. The expression of *PPM1M* is closely associated with patient prognosis, tumor immune checkpoint, tumor mutational burden, and microsatellite instability. *PPM1M* is closely associated with the infiltration of immune cells into the tumor microenvironment. In addition, *PPM1M* is involved in the regulation of several immune-related pathways.

**Conclusion:**

In pan-cancer, *PPM1M* affects patient prognosis and may be a potential immunological biomarker. Furthermore, *PPM1M* may be a potential therapeutic target in tumor immunology.

## 1. Introduction

Malignant tumors have become one of the leading causes of death in many regions. Cancer is a genetic disease. Tumor progression is associated with the tumor microenvironment (TME), where noncancerous cells are present within the tumor itself and are distributed around it. This has a strong influence on the biological behavior of the tumor genome.^[1]^ Of the various noncancerous cells, immune cells are key players in the development of tumors. Additionally, they may play an important role in the biological behavior of tumors. Among cellular immunological events, the most critical immune cells are CD4+ T cells, CD8+ T cells, and macrophages.^[2]^ The use of targeted immune checkpoint inhibitors (ICIs), most of which target programmed cell death protein 1 (PD-1), its ligand (PD-L1) and cytotoxic T-lymphocyte antigen-4, is continuously promoted in patients with cancer.^[3]^ A study showed that targeted therapy significantly improved the survival prognosis of patients with cancer; however, this treatment approach only benefited a subgroup of patients.^[4]^ Thus, there is a need to explore more effective and feasible strategies for improving patient outcomes. Hence, we focused on a pan-cancer analysis.

In the pan-cancer analysis, the gene of interest was studied, and the potential molecular mechanism of that gene in tumor progression was explored along with its correlation with the clinical prognosis of the patient. The development of technologies, such as whole genome sequencing, has allowed access to large amounts of genetic sequencing data and a large clinical database of several types of tumors from a comprehensive platform of multiple databases.^[5]^ This availability of large sample databases allows us to perform pan-cancer expression analysis. In human cancers, pan-cancer analysis can be used to identify commonalities and differences in clinical and epigenetic molecules by performing breadth analysis across multiple systems.^[6]^ In this study, pan-cancer analysis was performed to explore the role of the *PPM1M* gene in the development of malignancies.

PPM1M is a member of the metal-dependent protein phosphatase (PPM) family, which has not been explored much. The PPM family, also known as PP2C phosphatases, is one of 4 major families of eukaryotic protein serine/threonine phosphatases (PP1, PP2A, PP2B, and PP2C),^[7]^ whose active centers bind to manganese/magnesium ions and act as single subunit enzymes. There are 12 different classes of PPM phosphatase subunits in vertebrates. Phosphorylation is the most important posttranslational modification of proteins in response to cellular events. Of the different isoforms, specific regions of protein phosphatases and protein kinases are involved in a variety of cellular functions, such as the regulation of the cell cycle, cell differentiation, immune responses, and metabolic reactions. It does so by regulating reversible protein phosphorylation,^[7]^ and their functional inactivation or overactivation is associated with several different human diseases. It has been shown that members of the PPM family can inhibit the proliferation and oncogenic transformation of cancerous cells or even promote apoptosis, including PP2Cɛ and PP2Cδ.^[8]^ Knockout of *PPM1A* attenuated the trans activation of the human pregnane X receptor and promoted the proliferation of human hepatocellular carcinoma cells.^[9]^ A significant inhibitory effect on the proliferation and tumorigenicity of bladder malignancy cells exerted by the ectopic expression of PPM1B neutralizes the oncogenic effects of miR-186.^[10]^ In many cell types, PPM1D is associated with immune cell differentiation and responses. PPM1D regulates T cell maturation by activating p53.^[11]^ It is highly expressed in a variety of human tumors and is highly correlated with poor prognosis, for example, in esophageal carcinoma, lung cancer, ovarian epithelial cancer, and cervical cancer.^[12–14]^

Downregulation of the *PPM1M* gene in astrocytes significantly inhibits herpes simplex virus type 1 infection in the central nervous system (CNS). Astrocytes are important immune cells in the CNS, which may be due to phosphatase genes affecting the replication of herpes simplex virus type 1 or multiple signaling pathways regulating the immune response.^[15]^ Zi Ye et al^[16]^ suggested that *PPM1M* is a pivotal gene in the diagnosis of hepatocellular carcinoma, and it is a phosphoprotein phosphatase involved in RNA polymerization. However, the potential role of *PPM1M* in various types of tumors, the progression of human cancer, and evidence for human pan-cancer remain unclear. Given that the PPM family plays an irreplaceable role in cellular immune responses, the mechanism of action of *PPM1M* in cellular immune events is not yet clear. Based on the cancer genome atlas (TCGA) database, we performed pan-cancer expression analysis of *PPM1M* expression to determine the impact of *PPM1M* on the development and prognosis of cancer. Our findings suggest that *PPM1M* expression in various tumors correlates with patient prognosis and immune infiltration, suggesting that it might act as a potential prognostic immunotherapeutic biomarker.

## 2. Material and methods

### 2.1. Clinical sample collection

In this study, 20 paired bladder cancer and paracancerous tissues were collected from the Cancer Hospital of Guangxi Medical University. All patients were pathologically diagnosed with bladder cancer before tissue collection and were not treated with chemotherapy or radiotherapy. All the 20 patients signed an informed consent form.

This study was approved by the Ethics and Human Subjects Committee of Guangxi Medical University Cancer Hospital. All experiments and methods were performed according to the relevant guidelines and regulations.

### 2.2. Acquisition of data

RNA expression and clinical data from TCGA and genotype-tissue expression (GTEx) databases were sourced from the UCSC Xena database. DNA copy numbers were downloaded from the cBioPortal database.

### 2.3. Immunohistochemical stain

Cancerous and paracancerous tissue specimens were soaked in formalin and fixed. Each tissue was sliced to a thickness of 4 mm,^[17]^ embedded in wax blocks, and finally fixed on glass slides. Endogenous peroxidase activity was controlled at 37°C, suppressed, and stopped by incubation with 5% bovine serum albumin for 30 minutes. The slides were incubated with anti-PPM1M (1:300 concentration; 28,580, Signalway Antibody) at 4°C overnight and washed 3 times with phosphate buffered saline for 5 minutes each. The slides were incubated with the horseradish peroxidase secondary antibody at 37°C for 30 minutes. The sections were then incubated in phosphate buffered saline. After 3 washes, sections were incubated with diaminobenzidine. Finally, images were obtained using an optical microscope (Leica, Wetzlar, Germany).

### 2.4. Differential gene expression analysis

Sanger Box software (http://sangerbox.com/) was used to compare the differences in the expression of *PPM1M* between cancerous and paracancerous tissues from the TCGA database and normal samples from the GTEx database. Data were analyzed by plotting the differences in the expression of *PPM1M* using the tumor immune estimation resource 2 (TIMER2) database. Using the cBioPortal database, TCGA data analysis showed the mutation frequency, copy number variation (CNV), and mutation type of the *PPM1M* gene.

### 2.5. Survival analysis

To assess the prognostic value of *PPM1M* in patients with cancer, Kaplan–Meier analysis was used to show the difference in survival between patients in the high and low expression groups. The R packages used in this study were survival, glmnet, and survminer packages. Cox regression analysis was performed in R software (https://www.r-project.org/) using TCGA for each cancer type, overall survival (OS), disease-specific survival (DSS), and progression-free interval (PFI), to examine the relationship between *PPM1M* and OS, DSS, and PFI.

### 2.6. Immunological infiltration analysis

A correlation was found between *PPM1M* and immune infiltration using TIMER2 and CIBERSOFT methods. We focused on CD4+ and CD8+ T lymphocytes in our analysis. Estimates were used to analyze the relationship between *PPM1M* and immune, stromal, and estimated scores.

### 2.7. Gene enrichment analysis

To determine the pathways affected by *PPM1M*, gene set enrichment analysis (GSEA) was performed in the R package “cluster profiler.” The expression of *PPM1M* was divided into high *PPM1M* expression group and low *PPM1M* expression groups, and a list of sequenced genes was obtained based on the fold-change in mean gene expression between the 2 groups. The Kyoto encyclopedia of genes and genomes and HALLMARK pathways were used to evaluate biological processes.

### 2.8. Statistical analysis

Based on *PPM1M* mRNA expression values, the groups were divided into low and high *PPM1M* expression groups. Spearman correlation test was used to assess the relationship between *PPM1M*, tumor mutational burden (TMB) and microsatellite instability (MSI). Survival analysis was performed using Cox regression analysis to calculate hazard ratio (HR) values and log-rank *P* values. Statistical significance was set at *P* < .05.

## 3. Results

### 3.1. Comparison of the differences in the expression of *PPM1M* in normal and human tumor tissues

**Figure 1.**
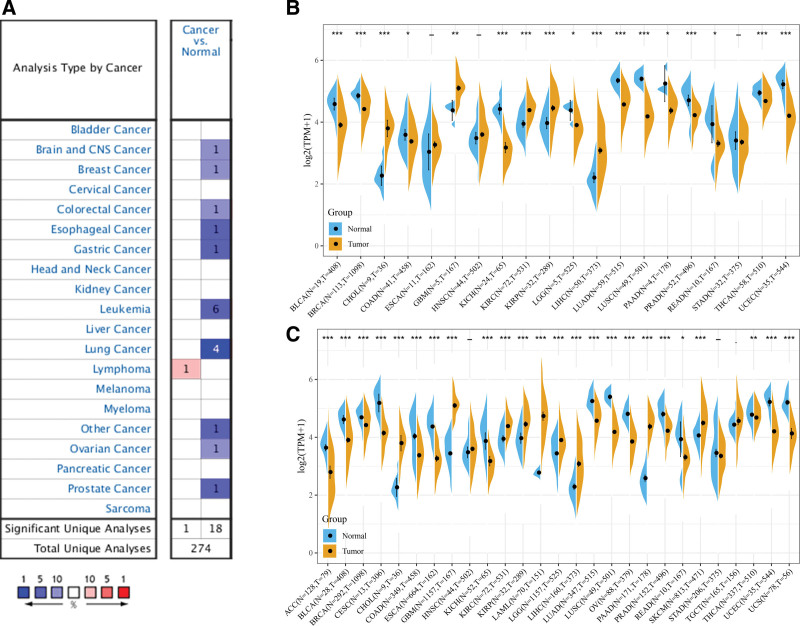
Analysis of the expression of *PPM1M* in pan-cancer. (A) Expression of *PPM1M* in tumor tissues and normal tissues were analyzed in the Oncomine database. (B) Differential expression of *PPM1M* in various tumor tissues and paraneoplastic tissues was analyzed in the TCGA database. (C) Integration of data from normal tissues from the genotype-tissue expression database and tumor tissues in the TCGA database to analyze the differences in the *PPM1M* expression in 27 tumors (*P < .05, ** *P* < .01, *** *P* < .001). TCGA = the cancer genome atlas.

Analysis of the Oncomine database showed that in most human normal tissues, including CNS, breast, colorectal, esophageal, gastric, ovarian, prostate, and lung tissues, *PPM1M* mRNA showed significantly high expression, whereas in malignant tissues, only lymphoma showed high expression (Fig. 1A). Sanger Box was used to determine the expression of *PPM1M* in 20 different types of tumors in the TCGA dataset. As shown in Figure 1B, *PPM1M* was expressed in bladder urothelial carcinoma (BLCA), breast invasive cancer (BRCA), cholangiocarcinoma (CHOL), kidney chromophobe (KICH), kidney renal clear cell cancer (KIRC), kidney renal papillary cell cancer (KIRP), liver hepatocellular cancer (LIHC), lung adenocarcinoma (LUAD), lung squamous cell carcinoma (LUSC), prostate adenocarcinoma (PRAD), thyroid cancer (THCA), and uterine corpus endometrial cancer (UCEC) at higher levels than in paired normal tissues (*P* < .001). The expression of *PPM1M* was downregulated in glioblastoma multiforme (GBM) (*P* < .01), colon adenocarcinoma (COAD), brain lower-grade glioma (LGG), pancreatic adenocarcinoma (PAAD), and rectal adenocarcinoma (READ) (*P* < .05). Low expression was also observed in esophageal cancer (ESCA), head and neck squamous cell cancer (HNSC), and stomach adenocarcinoma (STAD) (Fig. 1B). Because of the small sample size of normal tissues in TCGA database, we combined the data of the normal tissue from the GTEx database with the data of the tumor tissue from TCGA database and performed a differential analysis of *PPM1M* expression in 27 tumors. In this study, *PPM1M* was found to be overexpressed in 24 tumor types. These include the 17 tumor types mentioned earlier, along with the 7 others, such as adrenocortical carcinoma (ACC), cervical squamous cell carcinoma and endocervical adenocarcinoma (CESC), esophageal carcinoma (ESCA), acute myeloid leukemia (LAML), ovarian serous cystadenocarcinoma (OV), skin cutaneous melanoma (SKCM), uterine carcinosarcoma (UCS) (*P* < .05). In contrast, *PPM1M* was expressed at low levels in HNSC, STAD, and testicular germ cell tumors (TGCT) (Fig. 1C). Thus, the differences in the expression of *PPM1M* in different tumor types suggest that *PPM1M* has different regulatory mechanisms in different tumor types.

### 3.2. The relationship between the expression of *PPM1M* and clinical staging

**Figure 2.**
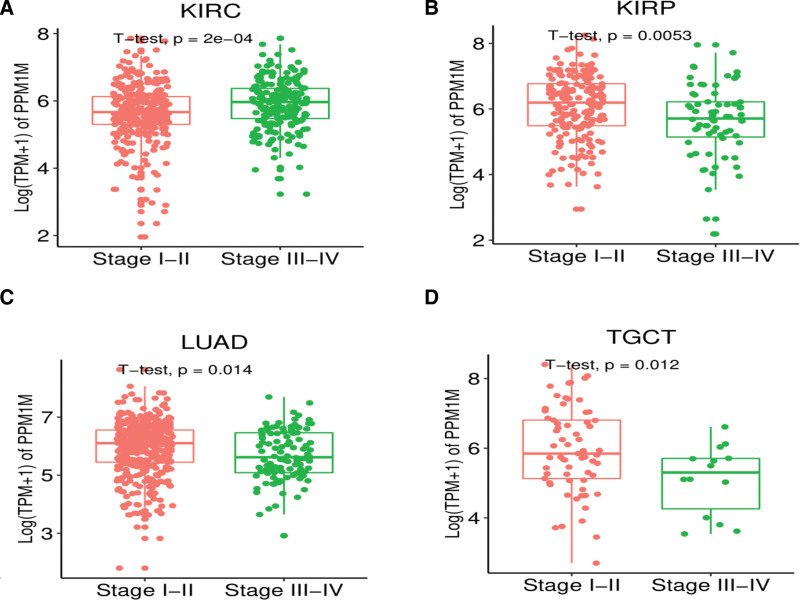
The expression of *PPM1M* in different clinical stages was analyzed in pan-cancer. (A–D) The differential expression of *PPM1M* in different stages of multiple tumors (*P* < .05 was considered statistically significant).

We continued to investigate the expression levels of *PPM1M* in a variety of tumors at different stages, and the results demonstrated remarkable differences in KIRC, KIRP, LUAD, and TGCT, with the expression levels of *PPM1M* in patients with cancer (stages I–II). Sample sizes for these types of tumors can be seen in Table S1, Supplemental Digital Content 1, http://links.lww.com/MD/I371. The expression level of *PPM1M* was significantly higher in patients in stages I to II than in those in stages III to IV (Fig. 2A–D). There was a increasing trend in the expression of *PPM1M* in mesothelioma (MESO) and READ, but the difference was not statistically significant. These and other types of tumors are shown in Figure S1, Supplemental Digital Content 2, http://links.lww.com/MD/I372.

### 3.3. Mutations in *PPM1M*

**Figure 3.**
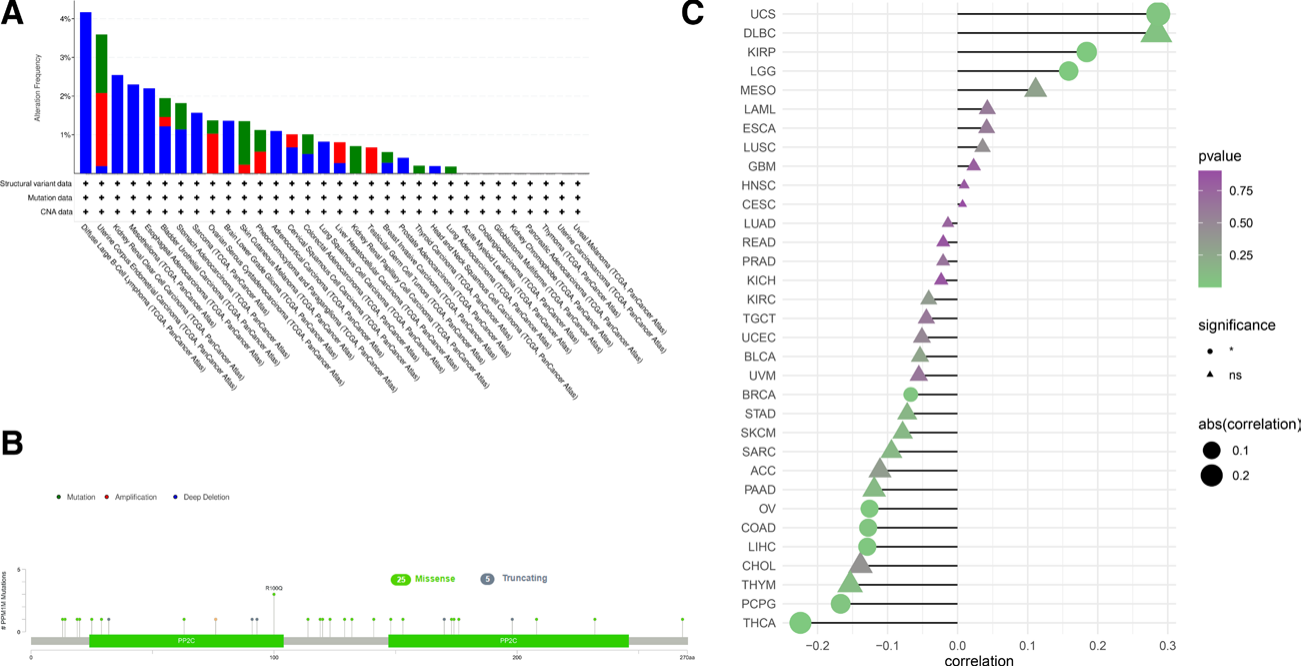
Genetic alterations in *PPM1M*. Using the cBioPortal database, we analyzed the mutational characteristics of *PPM1M*. (A) Mutation type, (B) frequency of mutation site changes, and (C) copy number alterations (*P < .05, NS: nonsignificant).

To investigate the relevance of *PPM1M* mutations in various human cancers, we extracted 33 tumor datasets from TCGA dataset using the cBioPortal database and examined *PPM1M* mutations. As shown in Figure 3A, deep deletions were the most common type of mutations in *PPM1M*, which was highest in diffuse large B-cell lymphoma (4.2%), whereas KIRC, MESO, and esophageal adenocarcinoma were almost more than 2%. The frequency of mutations was lowest in patients with LUAD (<0.5%). Additionally, mutations were present in almost all tumor types, whereas amplified mutations were present in certain tumor types. Figure 3B shows all data based on the type, locus, and case number of the *PPM1M* gene alterations, including missense, truncation, and fusion mutations, with PPM1M missense mutations being the predominant type of genetic alteration followed by truncation mutations. This study analyzed the expression of *PPM1M* copy number variants in different types of tumors, as shown in Figure 3C, which was positively correlated with *PPM1M* copy number in UCS, KIRP, and LGG, but negatively correlated with *PPM1M* copy number in BRCA, OV, COAD, LIHC, pheochromocytoma, paraganglioma (PCPG), and THCA.

### 3.4. Expression of *PPM1M* at the protein level

**Figure 4.**
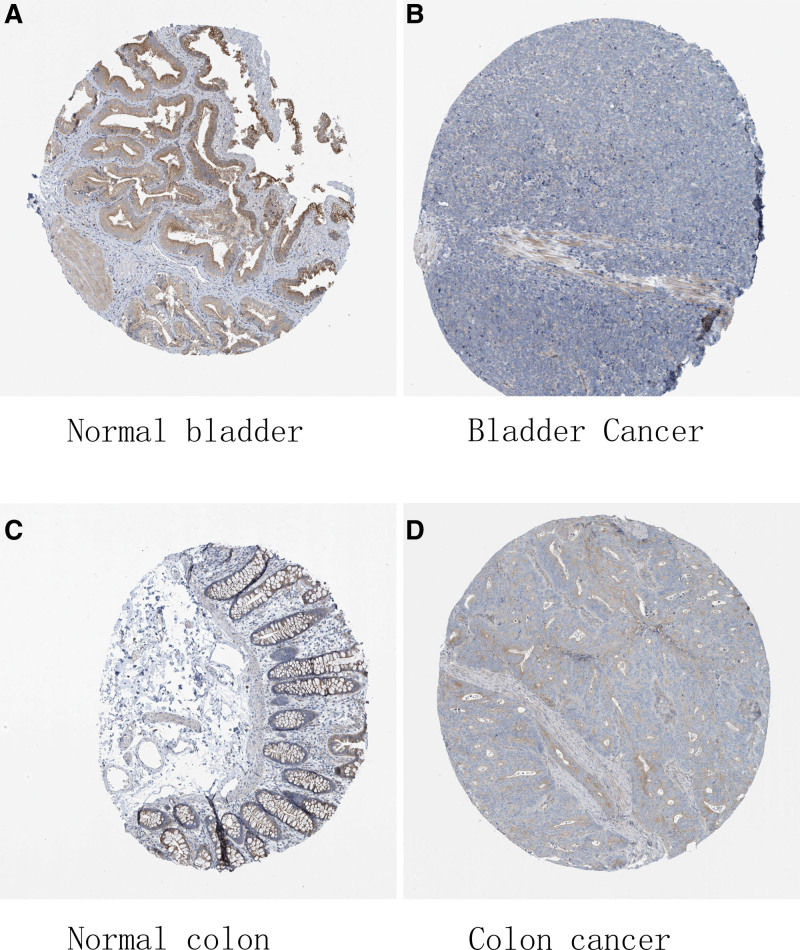
The protein levels of PPM1M in different tissues from the human protein atlas database: (A) normal bladder, (B) bladder cancer, (C) normal colon, and (D) colon cancer.

After determining the mutation type, the CNV and mRNA expression of *PPM1M* were analyzed. We assessed the protein expression level of *PPM1M* in tumors. Using the human protein atlas (HPA) database, the results suggested that the expression of *PPM1M* was high in the normal bladder and colon tissues, whereas the expression levels were significantly decreased in the bladder and colon cancer tissues (Fig. 4A–D).

**Figure 5.**
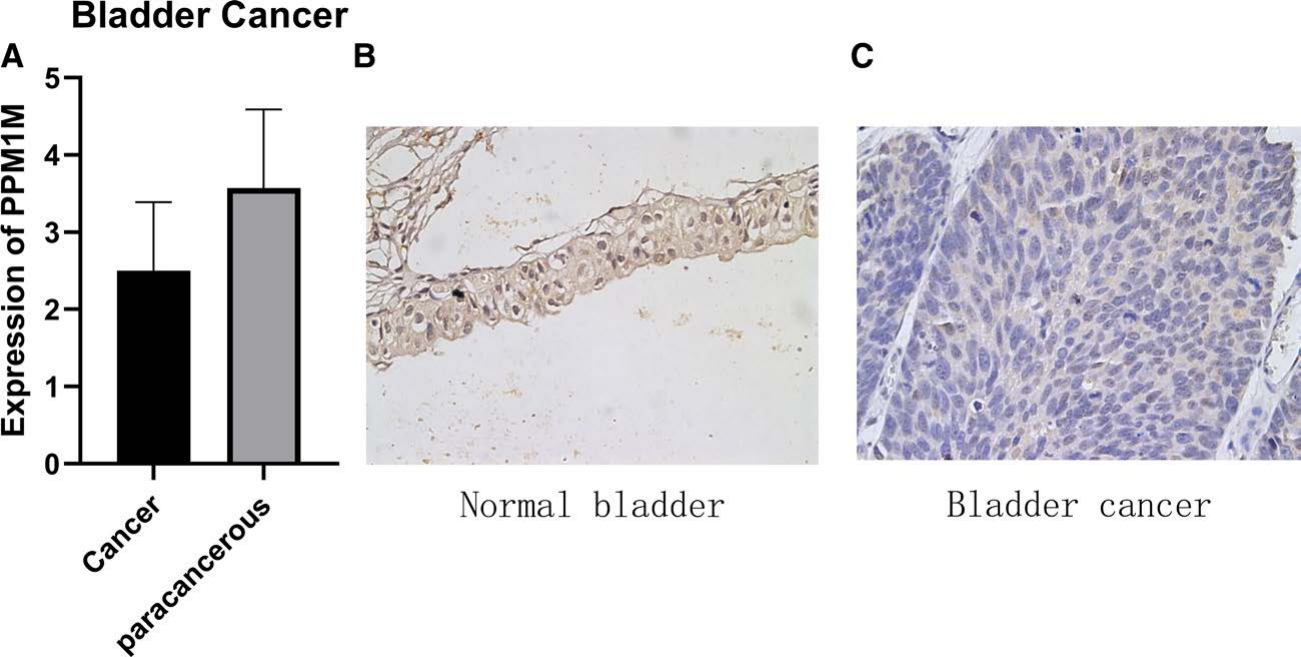
Images of immunohistochemical validation. (A) Analysis of PPM1M protein expression in bladder cancer using the human protein atlas database. (B) Bladder cancer para-cancer tissue results. (C) Bladder cancer tissue results.

Additionally, immunohistochemical validation of paired tissue specimens from patients with bladder cancer, in this study, showed that *PPM1M* was expressed at a significantly lower level in bladder cancer tissues than paired normal tissues (Fig. 5A–C), which was consistent with the results of the HPA database analysis.

**Figure 6.**
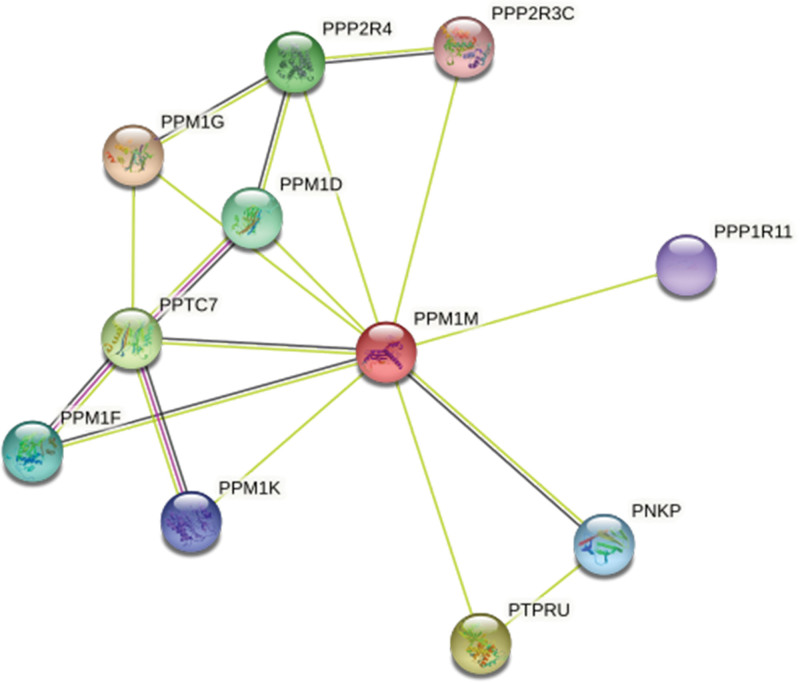
Construction of the protein-protein interaction network for *PPM1M*.

Moreover, we established a protein-protein interaction network, and the results showed that *PPM1M* was strongly connected to other proteins, including PPM1D, PPM1F, PPM1G, PPM1K, PPTC7, PPP2R3C, PPP2R4, PPP1R11, PNKP, and PTPRU (Fig. 6).

### 3.5. The prognostic impact of *PPM1M*

**Figure 7.**
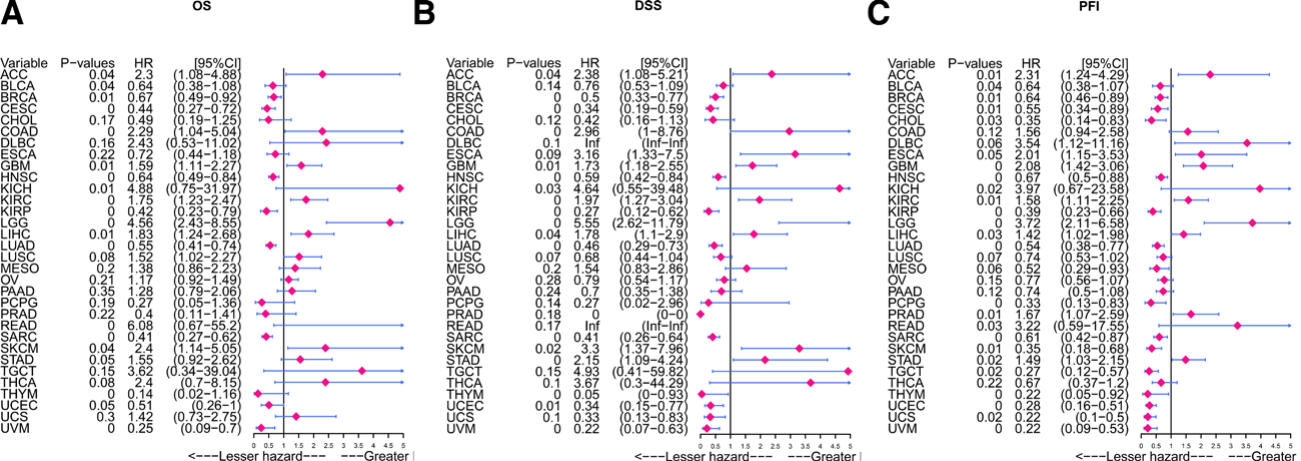
Forest map of the univariate Cox regression analysis. (A) Overall survival, (B) disease-specific survival, and (C) progression-free interval. The expression of *PPM1M* was significantly associated with prognosis in these types of cancer (*P* < .05) [hazard ratio (HR) > 1 indicates that *PPM1M* is a promoter of death].

To further study the effect of *PPM1M* on the prognosis of patients with cancer and OS, DSS, and PFI, we combined single-factor Cox regression analysis with Kaplan–Meier analysis. First, the *PPM1M* univariate Cox regression analysis of OS results suggested that *PPM1M* was a risk factor in ACC, COAD, GBM, KICH, KIRC, LGG, LIHC, READ, and SKCM (*P* < .05, HR > 1), but a favorable factor in BLCA, BRCA, CESC, HNSC, KIRP, LUAD, sarcoma (SARC), thymoma (THYM), and uveal melanoma (UVM) (*P* < .05, HR < 1) (Fig. 7A). DSS results revealed that *PPM1M* was an unfavorable element for ACC, COAD, GBM, KICH, KIRC, LGG, LIHC, SKCM, and STAD (*P* < .05, HR > 1), but an favorable element for BRCA, CESC, HNSC, KIRP, LUAD, SARC, THYM, UCEC, and UVM (*P* < .05, HR < 1) (Fig. 7B). The PFI results indicated that *PPM1M* was hazardous for ACC, GBM, KICH, KIRC, LGG, LIHC, READ, PRAD, and STAD (*P* < .05, HR > 1), but safe for BLCA, BRCA, CESC, CHOL, HNSC, KIRP, LUAD, PCPG), SARC, THYM, UCEC, UCS, and UVM (*P* < .05, HR < 1) (Fig. 7C).

**Figure 8.**
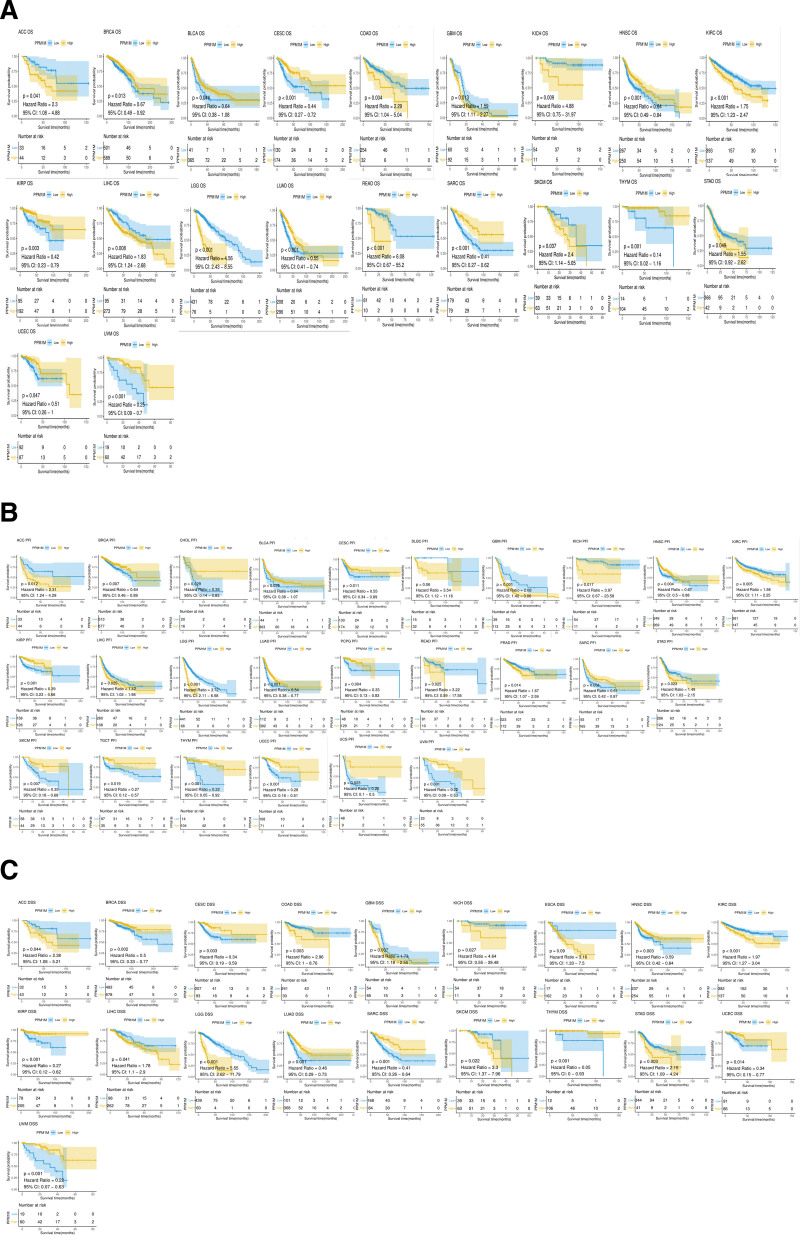
The Kaplan–Meier survival analysis of *PPM1M* in multiple types of tumors. (A) Overall survival, (B) disease-specific survival, and (C) progression-free interval. Tumor types with *P* < .05 are shown. *PPM1M* was significantly associated with the prognosis for these types of tumors (*P* < .05) (|HR| > 1 indicates that *PPM1M* is a promoter of death).

Second, Kaplan–Meier analysis of OS indicated that *PPM1M* was an favorable factor for BRCA, BLCA, CESC, KIRP, SARC, THYM, UVM, HNSC, LUAD, and UCEC (*P* < .05, HR < 1); however, it was an unfavorable factor for ACC, COAD, GBM, KICH, KIRC, LIHC, LGG, READ, SKCM, and STAD (*P* < .05, HR > 1) (Fig. 8A). DSS analysis showed that *PPM1M* was safe for BRCA, CESC, HNSC, KIRP, LUAD, SARC, THYM, UCEC, and UVM (*P* < .05, HR < 1); however, it was hazardous for ACC, COAD, GBM, KICH, KIRC, LIHC, LGG, SKCM, and STAD (*P* < .05, HR > 1)(Fig. 8B). PFI analysis showed that *PPM1M* was an favorable contributor to BRCA, SKCM, CHOL, BLCA, CESC, HNSC, TGCT, KIRP, LUAD, PCPG, SARC, THYM, UCEC, UCS, and UVM (*P* < .05, HR < 1); however, it was an unfavorable contributor to ACC, GBM, KICH, KIRC, LIHC, LGG, READ, PRAD, and STAD (*P* < .05, HR > 1) (Fig. 8C).

### 3.6. Gene set enrichment analysis of *PPM1M*

**Figure 9.**
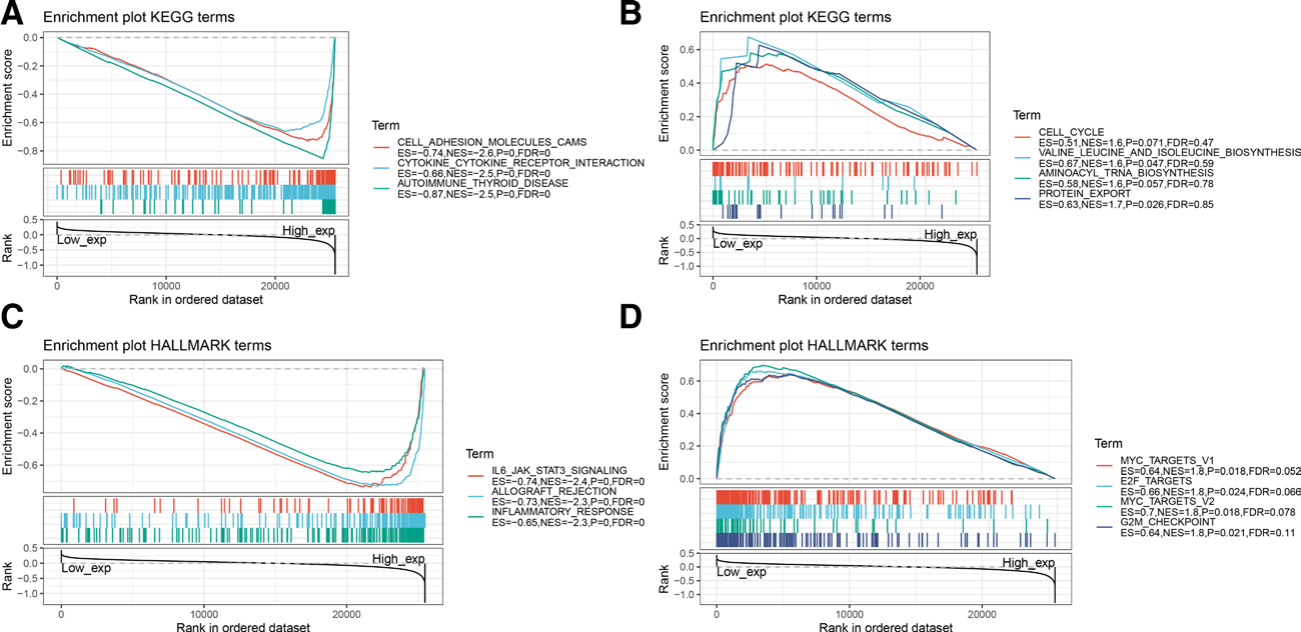
Gene set enrichment analysis results for the *PPM1M* high and low expression groups. (A) Enrichment analysis of the Kyoto Encyclopedia of Genes and Genomics (KEGG) pathway in the *PPM1M* high expression group. (B) Enrichment analysis of the KEGG pathway in the *PPM1M* low expression group. (C) Analysis of the hallmark pathway in the high *PPM1M* expression group. (D) Hallmark pathway analysis of the *PPM1M* low expression group. Only a few major gene sets are shown in the figure (only NOM *P* < .05 was considered statistically significant). KEGG = Kyoto encyclopedia of genes and genomes.

We further explored the possible signaling pathways involved in PPM1M. We used GSEA to identify the functional enrichment of PPM1M high expression and PPM1M low expression to assess the role of PPM1M in the TCGA database of 33 tumors (Fig. 9). The results showed that high PPM1M expression was highly correlated with immune-related pathways, including cell adhesion molecules, cytokine receptor interactions, and autoimmune thyroid disease. *PPM1M* is strongly linked to the regulation of the immune microenvironment in cancer and ligand-receptor interactions in immune cells. Significantly enriched pathways with low *PPM1M* expression included, valine, leucine, and isoleucine biosynthesis, and protein export, which may affect tissue cell metabolism and its cycle (Fig. 9A and B).

The results of HALLMARK pathway analysis showed that *PPM1M* was correlated with interleukin-6 (IL-6) JAK stat3 signaling, allograft rejection, and inflammatory response, suggesting that *PPM1M* is closely related to immunoregulation. The pathways that were significantly enriched when *PPM1M* was lowly expressed included MYC targets V1, E2F targets, MYC targets V2, and G2M checkpoint, suggesting its involvement in tumorigenesis and development (Fig. 9C and D).

### 3.7. Relationship between *PPM1M* and immune infiltration

**Figure 10.**
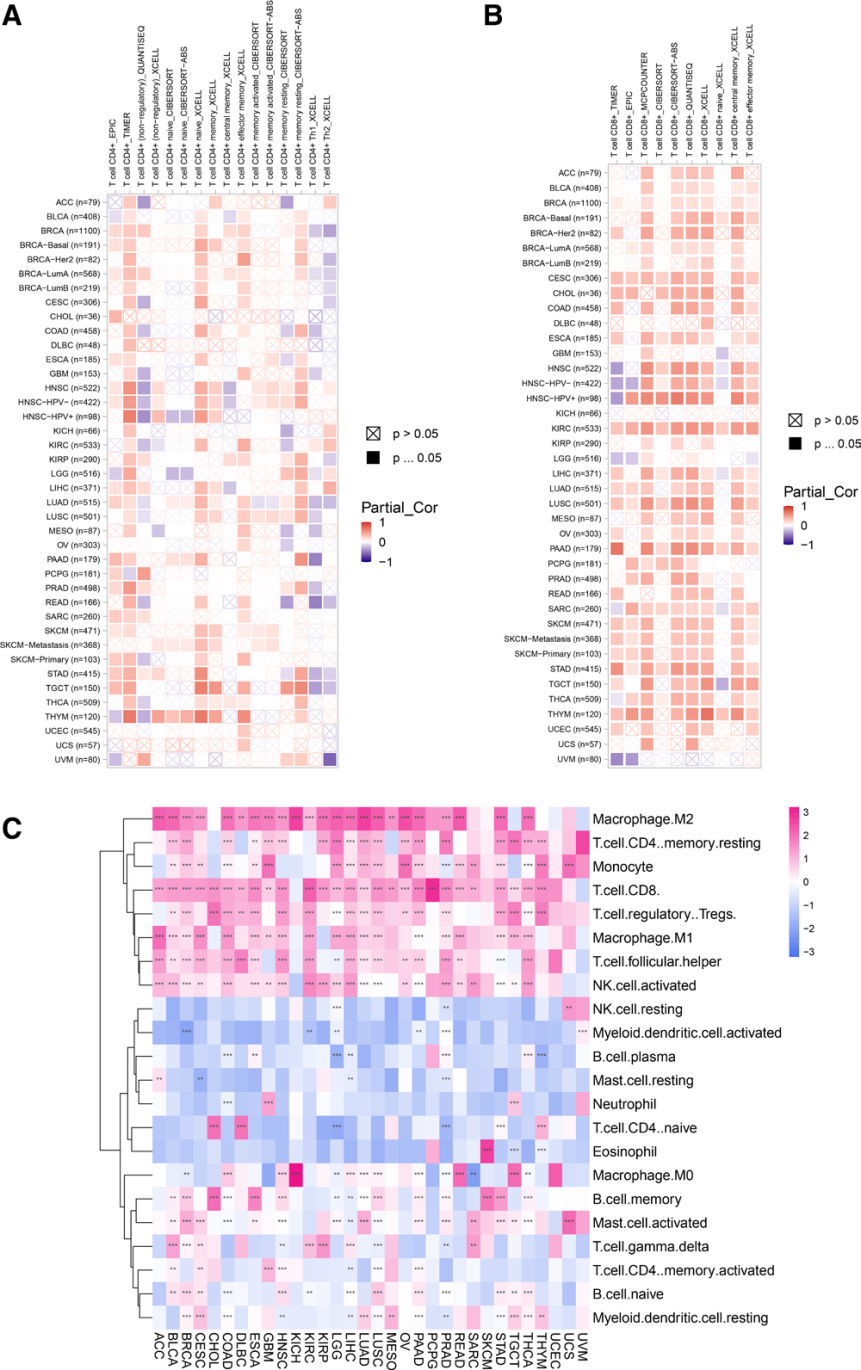
The correlation analysis of *PPM1M* and immune cell infiltration in the tumor immune estimation resource 2 database. (A) Results of the analysis of *PPM1M* and CD4+ T cells. (B) Results of the analysis of *PPM1M* and CD8+ T cells. (C) Analytical results of *PPM1M* and the indicated immune cells. Red indicates a positive correlation; blue indicates a negative correlation and color depth is proportional to the correlation. *P < .05; **P < .01; ***P < .001 and ****P < .0001.

To understand the relationship between *PPM1M* and immune infiltration, in this study, we explored immune infiltration data from 2 origins. The outcomes indicated that *PPM1M* was actively associated with the infiltration levels of CD8+ T lymphocytes and CD4+ T lymphocytes in TCGA but had a negative correlation with the infiltration levels of nonregulatory CD4+ T lymphocytes (Fig. 10A and B). The correlation between *PPM1M* and the 22 types of immune cells was assessed using the CIBERSOFT method, which showed that *PPM1M* was positively correlated with the level of infiltration of Tregs, CD8+ T lymphocytes, and M2-like macrophages; however, it had a negative correlation with the level of infiltration of naive CD4+ T cells (Fig. 10C).

In conclusion, the results of the analysis of the 2 types of immune cell infiltration are consistent. This suggests that *PPM1M* enhances T cell infiltration, which could explain its protective role in most types of cancer.

### 3.8. Correlation of *PPM1M* with immune regulation-related genes and chemokines

To explore the potential association between *PPM1M* and immune-related factors, Figure S2A–C, Supplemental Digital Content 3, http://links.lww.com/MD/I373 summarizes the relationships between *PPM1M* and estimated immune, stromal, and estimated scores (*P* < .005). The outcome showed that *PPM1M* was significantly correlated to the estimated immune scores of multiple carcinomas: BLCA, BRCA, CESC, CHOL, COAD, DLBC, ESCA, GBM, HNSC, KIRC, KIRP, LAML, LGG, LIHC, LUAD, LUSC, OV, PAAD, PRAD, READ, SARC, SKCM, STAD, TGCT, THCA, THYM, UCEC, and UCS. Second, *PPM1M* was significantly correlated with stromal scores for various cancers, including ACC, BLCA, BRCA, CESC, CHOL, COAD, ESCA, GBM, HNSC, KIRC, LAML, LGG, LIHC, LUAD, LUSC, OV, PAAD, PRAD, READ, SKCM, STAD, TGCT, THCA, UCEC, and UCS. Finally, *PPM1M* was significantly correlated with the estimated scores for multiple cancers, including ACC, BLCA, BRCA, CESC, CHOL, COAD, DLBC, ESCA, GBM, HNSC, KIRC, KIRP, LAML, LGG, LIHC, LUAD, LUSC, OV, PAAD, PRAD, READ, SKCM, STAD, TGCT, THCA, THYM, UCEC, and UCS. In conclusion, these results suggest that *PPM1M* may be involved in immune regulation and recruitment of cellular tropism.

**Figure 11.**
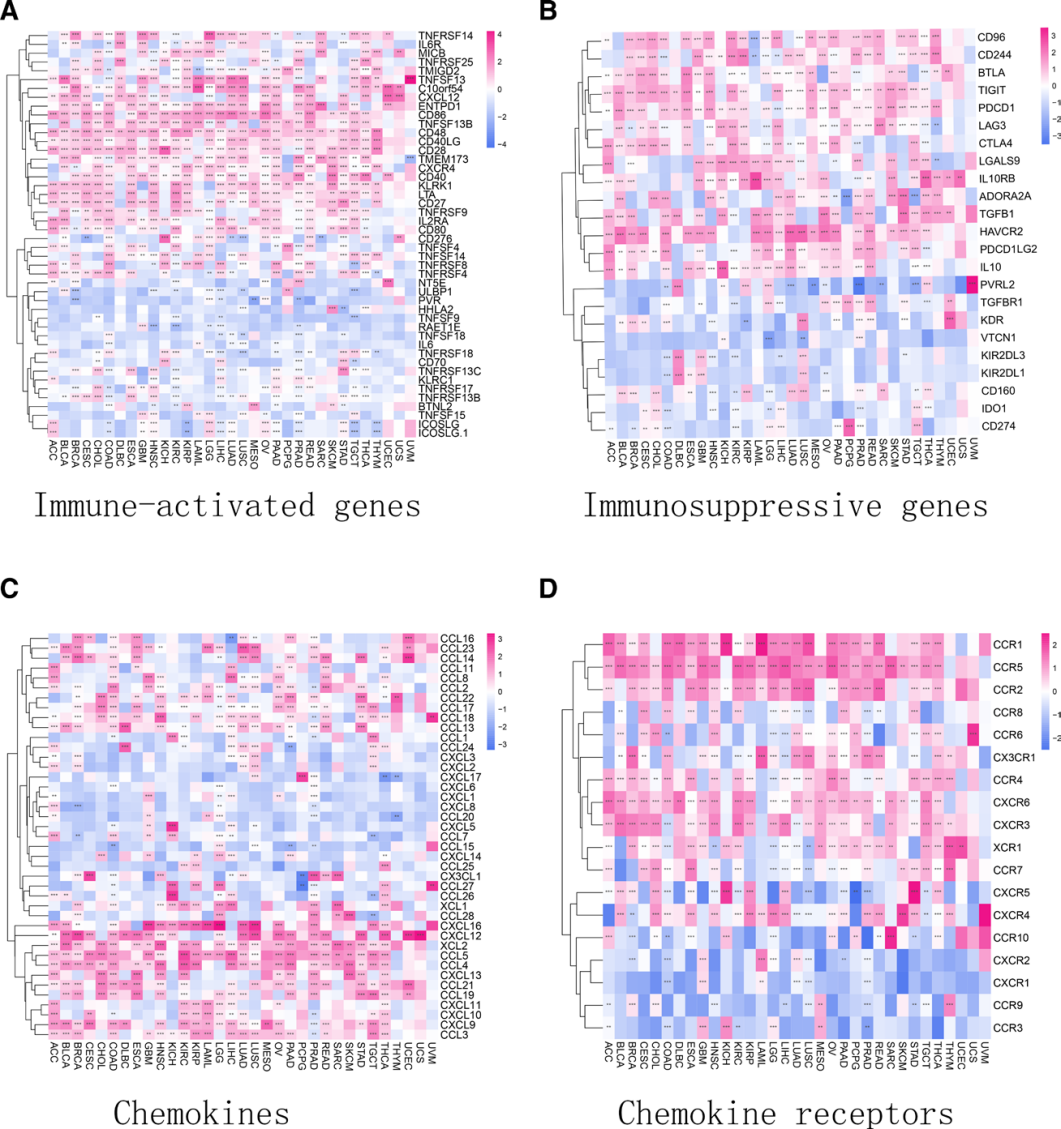
Results of the correlation analysis between *PPM1M* and immunoregulatory genes represented by a heat map. (A) Relationship between *PPM1M* and immune activating genes. (B) Relationship between *PPM1M* and immunosuppressive genes. (C) Relationship between *PPM1M* and chemokine genes. (D) Relationship between *PPM1M* and chemokine receptor genes. *P < .05; **P < .01; ***P < .001 and ****P < .0001.

T cell depletion is the loss of T cell function observed in patients with chronic infectious diseases or cancer. T cells are abundant in cancer patients; however, most of their functions are lost. We explored the links between *PPM1M* and immune activation genes, immune hindrance genes, chemokines, and the corresponding chemokine receptors in depleted T cells. The results suggested that *PPM1M* was positively associated with immune-activating and immune-suppressing genes of depleted T cells in pan-cancer, including ENTPD1, CD48, CD28, CD86, HAVCR2, TIGIT, PDCD1, LAG3, CTLA4, and CD96 (Fig. 11A and B). Additionally, among chemokines and chemokine receptors, *PPM1M* was positively associated with chemokines such as CXCL16, CXCL12, XCL2, CCL3, CCL4, and CCL5. A similar relationship was observed with the corresponding chemokine receptors, such as CCR1, CCR2, CCR4, and CCR5 (Fig. 11C and D).

### 3.9. Expression of *PPM1M* in human tumors with the immune checkpoint (ICP) gene

**Figure 12.**
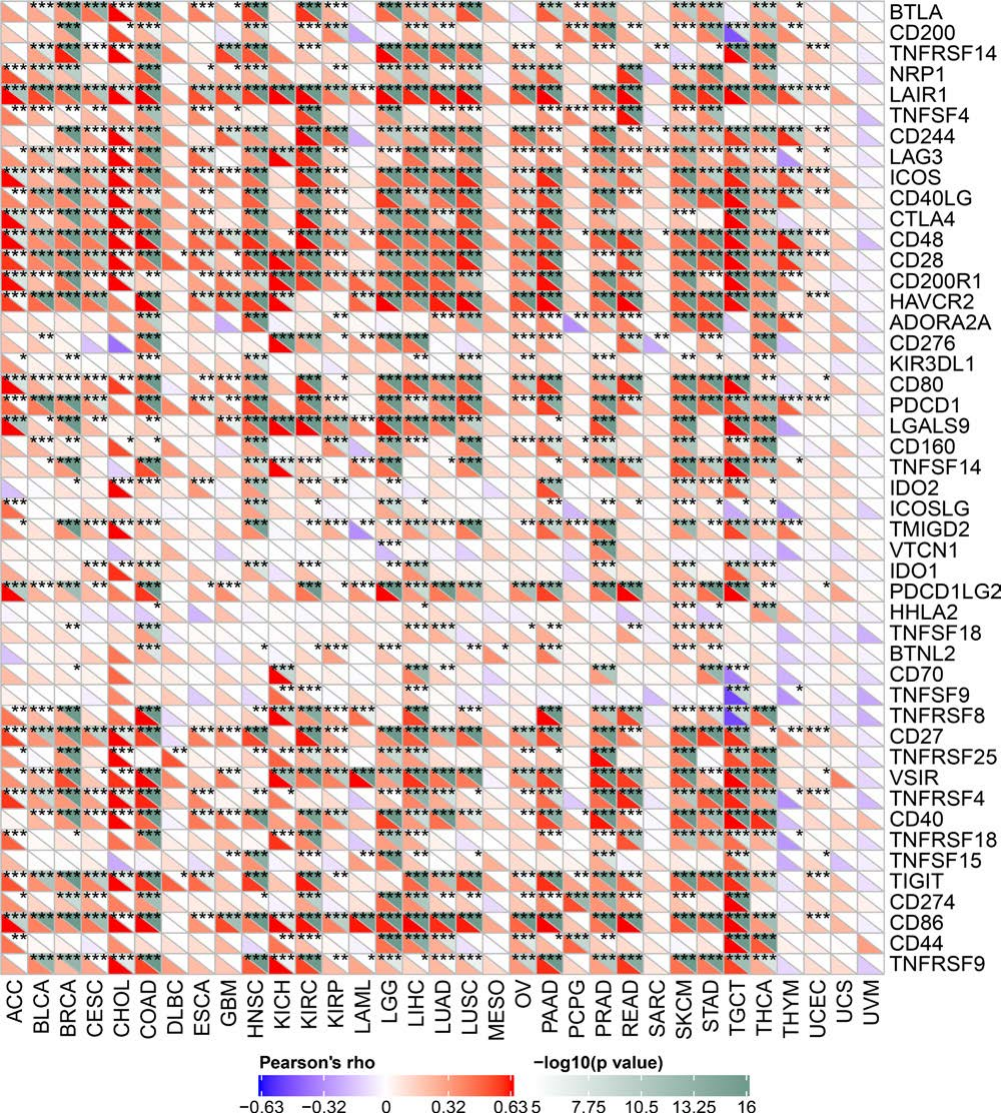
Relationship between *PPM1M* and immune checkpoint genes in pan-cancer. *P < .05; **P < .01; ***P < .001.

The ICP gene affects immune cell infiltration and has a significant impact on immunotherapy efficacy. Therefore, to explore the potential role of *PPM1M* in immunological therapy, we investigated the relationship between the expression of *PPM1M* and ICP (Fig. 12). The results showed that among the many ICP genes, the expression of *PPM1M* was closely associated with multiple tumors. This suggests that *PPM1M* has different effects on ICP through different routes and that *PPM1M* has the potential to be an effective immunological therapeutic target. First, *PPM1M* was actively related to ICP genes in COAD, TGCT, PRAD, KIRC, LIHC, HNSC, PAAD, LGG, and BRCA, suggesting that immunological treatment may target ICP genes when *PPM1M* is highly expressed. Second, *PPM1M* was inactively related to ICP genes in MESO, UVM, and UCS, indicating that immunological treatment targeting ICP genes may be ineffective when *PPM1M* is highly expressed. Thus, *PPM1M* may hold promise as a potential pan-cancer biomarker or as a novel immunological therapeutic target when used to predict immunotherapeutic responses or achieve a desired therapeutic outcome, respectively.

### 3.10. Association of *PPM1M* with tumor mutational burden and microsatellite instability

**Figure 13.**
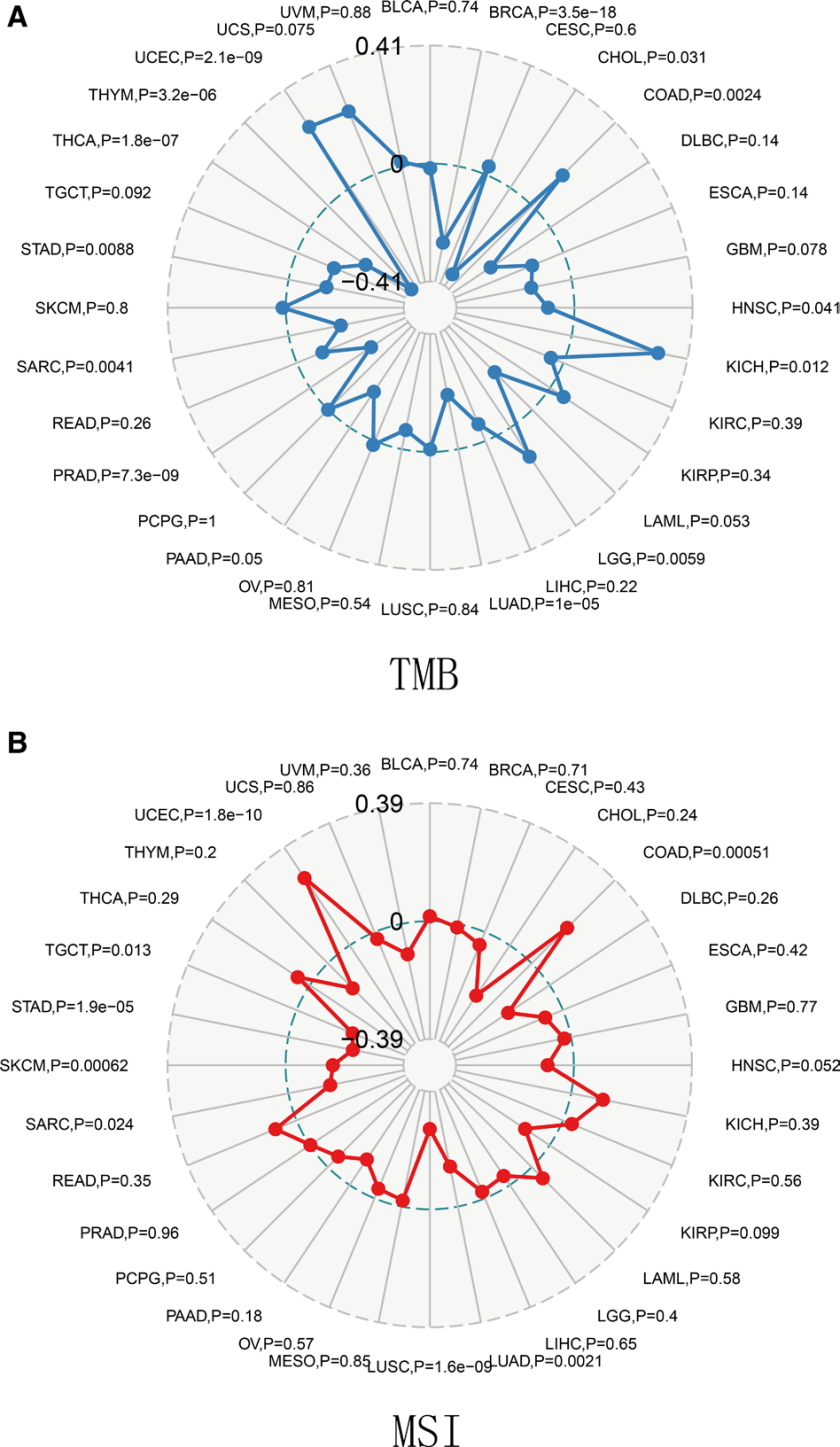
Relationship between *PPM1M* and tumor mutational burden (TMB), microsatellite instability (MSI) in multiple cancers. (A) TMB and (B) MSI. MSI = microsatellite instability, TMB = tumor mutational burden.

TMB and MSI can be used as valid prognostic biomarkers and indicators of response to immunotherapy in a wide range of cancers.^[18,19]^ TMB is a quantifiable biomarker used to reflect the number of mutations in tumor cells, and MSI is a novel microsatellite allele that emerges in tumors. In the present study, we explored the correlation between *PPM1M* and novel dynamic biomarkers of immune checkpoint inhibitors (TMB and MSI). The results showed that *PPM1M* was significantly and actively related to TMB in COAD, KICH, LGG and UCEC. However, *PPM1M* was negatively correlated with TMB in BRCA, CHOL, HNSC, LUAD, PRAD, SARC, STAD, THCA, and THYM (Fig. 13A). In contrast, the expression of *PPM1M* was positively related to MSI in COAD and UCEC, but negatively correlated with MSI in LUAD, LUSC, SARC, SKCM, STAD, and TGCT (Fig. 13B).

## 4. Discussion

*PPM1M* is a member of the PPM family. PPM phosphatase regulates a variety of cellular functions such as cell cycle regulation, cell differentiation, immune response, and cell metabolism. Mutation, overexpression, or deletion of the PPM phosphatase gene can lead to abnormal cellular responses, resulting in various human diseases. It has been shown that *PPM1M* accumulates in the nucleus and functions in association with other members of the PPM family, such as PPM1A and PPM1B, which selectively act on IKKβ downstream of TAK1 to inhibit the IL-1-induced activation of NF-κB.^[20]^ Activation of NF-κB occurs through 2 distinct signaling pathways, the classical and nonclassical NF-κB pathways, which are usually triggered by inflammatory, pathogenic, or stress signals. NF-κB has been studied in various cancers, which are triggered and developed as a result of the dysregulation of individual subunits or whole pathways. By expressing PD-L1, tumor cells are often able to evade cell death induced by surrounding T cells. Upregulation of nonclassical NF-κB has been observed in tumor dendritic cells, improving the synergistic relationship between dendritic cells and CD8+ T cells.^[21]^ RelA, a transcription factor downstream of the classical NF-κB pathway, either increases the resistance of cancer cells to T cell-induced cell death or increases the expression of PD-L1 in ovarian cancer cells.^[22,23]^ Although previous studies have suggested that the members of the PPM family may regulate the NF-κB pathway to influence immunity; the relationship between *PPM1M* and immunity in pan-cancer is not yet clear. Therefore, this is the first pan-cancer study conducted on the *PPM1M* gene.

In this study, we investigated the expression of *PPM1M* in pancytopenia and its prognostic significance. First, the differences in the expression of *PPM1M* in tumor tissues and paired normal tissues were compared, and *PPM1M* mRNA expression levels were found to be high in 8 normal tissues, including brain and CNS, breast, colorectal, esophageal, gastric, ovarian, prostate, lung, and leukemia. Among malignant tissues, only lymphoma showed high expression, whereas the others were either low or absent. Second, the results of testing the TCGA dataset suggested that the expression levels of *PPM1M* were significantly different in different tumor types. Meanwhile, the data of the normal tissue from the GTEx database were integrated with the data of the tumor tissue from TCGA database, and joint analysis revealed that *PPM1M* was overexpressed in 24 tumors. Furthermore, in-depth exploration of the *PPM1M* protein in stages I to II and III to IV of pan-cancer revealed that the expression levels in stages I to II were greater than those in stages III to IV in KIRP, LUAD, and TGCT. However, a decreasing trend in expression was observed for ACC, BLCA, MESO, and READ. Among the types of genetic alterations, *PPM1M* had the highest frequency of profound deletions, occurring most frequently in diffuse large B-cell lymphoma and least frequently mutated in patients with LUAD. CNV of *PPM1M* differed in pan-cancer with UCS, KIRP, and LGG positively correlated with *PPM1M* copy number, whereas BRCA, OV, COAD, LIHC, PCPG, and THCA were negatively correlated with *PPM1M* copy number. In this study, immunohistochemistry was used to examine the expression levels of *PPM1M* in bladder cancer and its paracancerous tissues suggesting that *PPM1M* was highly expressed in the paracancerous tissues of bladder cancer, while the expression levels decreased significantly in bladder cancer. These results were consistent with those of the HPA database analysis of *PPM1M* protein expression levels in bladder cancer.

Additionally, we found that the expression of the *PPM1M* gene plays a different role in tumor prognosis in different types of tumors. The results of the univariate Cox regression analysis of OS, DSS, and PFI were similar to those of Kaplan–Meier analysis. Taken together, these results indicate that *PPM1M* may act as a protective factor in a variety of tumors when expressed at high levels. Therefore, *PPM1M* may act as a protective factor in humans; however, its mechanism is still unknown and requires further research.

Further, we used GSEA analysis to investigate the results of the different levels of *PPM1M* expression involved in regulating multiple pathways and found that when *PPM1M* is highly expressed, it is closely associated with immune-related pathways and that the IL-6/JAK/STAT3 pathway plays a key role in the pathogenesis and progression of malignant tumors. Elevated IL-6 levels are commonly observed in patients with chronic inflammation. Moreover, elevated levels of IL-6 induce excessive activation of JAK/STAT3 signaling, which drives the proliferation, survival, invasion, and metastasis of malignant cells. Furthermore, it induces an immunosuppressive tumor microenvironment, resulting in a strong suppression of anti-tumor immune responses, consistent with the critical role of the JAK/STAT signaling pathway in chronic inflammation and inflammation-mediated progression of cancer,^[24–26]^ which is often associated with poor patient prognosis. Additional evidence suggests that inhibition of IL-6/JAK/STAT3 signaling may enhance the antitumor efficacy of ICIs. The inhibition of STAT3 activity has been shown to improve the survival prognosis of patients with prostate, colon, and pancreatic cancers.^[27]^

During cancer immunosurveillance, the immune system recognizes and destroys new tumor cells. Therefore, cancer immunosurveillance is essential for eliminating carcinoma cells. Cytotoxic CD8+ T lymphocytes are a population of cells that are designed to kill infected cells. CD8+ T cells play an important role in antitumor immune processes.^[28,29]^ Cytotoxic CD8+ T cells depend on CD4+ T cells to play a paracrine role in the initiation and memory processes.^[30]^ In this study, we used 2 methods to analyze immune cell infiltration data, and the results showed that *PPM1M* has a positive association with CD8+ T lymphocytes, CD4+ T lymphocytes, and natural killer (NK) cells. This finding explains the protective role of *PPM1M* against most malignancies.

Previous analyses have shown that *PPM1M* is closely associated with immune pathways. It is known to be associated with cell adhesion molecules, which is dynamically regulated in response to changes in the tissue and its interstitial matrix caused by tumor growth. Additionally, it plays a central role in mediating the growth of tumor vessels,^[31]^ and it has been shown that CAM determines the metastatic behavior of cancer cells. When CAM is highly expressed, patients with endometrial cancer may have a significantly poorer OS and relapse-free survival (RFS).^[32]^ Thus, *PPM1M* might play an inhibitory role in the CAM pathway when expressed at high levels, which is consistent with the findings of this study, where *PPM1M* plays a protective role in some cancers. Tregs help malignant cells escape cytotoxic CD8+ T cell populations during tumor immunization.^[33,34]^ These results suggest that *PPM1M* was positively correlated with the level of Treg infiltration, indicating that although cytotoxic CD8+ T lymphocytes were abundant, their functions were restricted. Additionally, *PPM1M* is positively associated with NK cells and negatively associated with naive CD4+ T cells, suggesting that *PPM1M* may directly or indirectly influence the process by which NK cells function.

Patients with chronic infectious diseases and cancers have specific CD8+ T lymphocytes that are under constant antigen stimulation, which can lead to a dysfunctional state of T cells called “depletion.” Therefore, the loss of T cells is also known as T cell depletion. Cytotoxic CD8+ T cells are the main immune cells that control and clear tumor cells. Patients with cancer usually have a large number of T cells, which are often not at an optimal state due to immune tolerance and immunosuppressive mechanisms, and differentiate into a dysfunctional state called “depletion.”^[35]^ In this study, *PPM1M* was found to be positively associated with some of the immune-activating and immune-suppressing genes that deplete T cells in pan-cancer, such as ENTPD1, CD48, CD28, CD86, HAVCR2, TIGIT, PDCD1, LAG3, CTLA4, and CD96. Additionally, *PPM1M* positively correlated with a variety of chemokines and chemokine receptors. In particular, there was a significant positive correlation among CCL4, CCL5, CCR4, and CCR5. These results confirm the possible role of *PPM1M* in ICP. A growing number of studies support the fact that the application of programmed death receptor-1/ligand 1 (PD-1/L1) antibodies is effective in prolonging the survival of patients with cancer and can induce prolonged remission of malignant tumors.^[36]^ The effective application of PD-1/PD-L1 pathway blockade highlights the key role played by immunosuppressive receptors in tumor progression.

This study also found that in most human cancers, *PPM1M* positively correlated with immune, mesenchymal, and estimated TME scores, but the expression of *PPM1M* in human cancers did not always positively correlate with tumor-infiltrating lymphocytes, suggesting that *PPM1M* plays different immunomodulatory roles in different types of cancer. In this study, the correlation of *PPM1M* with TMB and MSI demonstrated that in malignant tumors, *PPM1M* is closely associated with the TMB. TMB and MSI serve as novel dynamic biomarkers for ICIs, and TMB predicts checkpoint blockade responses in most tumors. Goodman concluded that patients with microsatellite (stable/high) or TMB tumors have a longer median progression-free survival and are more likely to benefit from immunotherapy.^[37]^ However, the mechanism of action of *PPM1M* in TMB and MSI requires further research.

The limitation of our study is that more in vivo targeting of the antitumor activity of *PPM1M* and additional clinical trials are required to validate the immune checkpoint action of *PPM1M*.

## 5. Conclusion

Targeting the *PPM1M* gene has shown considerable promise for antitumor immunity. Additional clinical studies on tumor-based immunity are required to maximize the utility and effectiveness of *PPM1M* gene-based predictive biomarkers and to use them in precision medicine for patients with cancer.

## Acknowledgments

The authors are grateful to all patients for their active participation in this study and Guangxi Zhuang Autonomous Region People’s Hospital Youth Fund (QN2021-11).

## Author contributions

**Conceptualization:** Rongruo Zeng, Lulu Wang, Ye Yang, Jie Yang.

**Data curation:** Rongruo Zeng, Lulu Wang.

**Formal analysis:** Rongruo Zeng, Lulu Wang, Yuxu Zhang, Ye Yang.

**Methodology:** Rongruo Zeng, Lulu Wang, Yuxu Zhang, Ye Yang, Jie Yang.

**Software:** Lulu Wang.

**Supervision:** Jie Yang, Yan Qin.

**Validation:** Yuxu Zhang, Ye Yang.

**Visualization:** Yuxu Zhang.

**Writing – original draft:** Rongruo Zeng.

**Writing – review & editing:** Yan Qin.

## Supplementary Material






