# Supplementary material for: Exploring the immunological role and prognostic potential of PPM1M in pan-cancer
Source: Medicine (Baltimore). 2023 Mar 24;102(12):e32758. doi: 10.1097/MD.0000000000032758 (PMC10036021; doi:10.1097/MD.0000000000032758)
Supplement: Supplementary file 1 [file medi-102-e32758-s001.pdf]

supplementary table 1: the table lists the abbreviations and full names of some tumors, as well as the number of tumor group samples and normal group samples.

Abbreviations	Full names of the various tumors	Normal group samples(N)	Tumor group samples(T)
KIRC	kidney renal clear cell cancer	72	531
KIRP	kidney renal papillary cell cancer	32	289
LUAD	lung adenocarcinoma	57	515
TGCT	testicular germ cell tumors	165	156
LIHC	liver hepatocellular cancer	50	737
LUSC	lung squamous cell carcinoma	49	501
PAAD	pancreatic adenocarcinoma	4	178
READ	rectal adenocarcinoma	10	167
SKCM	skin cutaneous melanoma	813	471
STAD	stomach adenocarcinoma	206	375
THCA	thyroid cancer	337	510
