# Supplementary material for: Exploring the immunological role and prognostic potential of PPM1M in pan-cancer
Source: Medicine (Baltimore). 2023 Mar 24;102(12):e32758. doi: 10.1097/MD.0000000000032758 (PMC10036021; doi:10.1097/MD.0000000000032758)

Supplementary Figure 1: The decreasing trend of *PPM1M* expression in different WHO stages of pan-cancer.

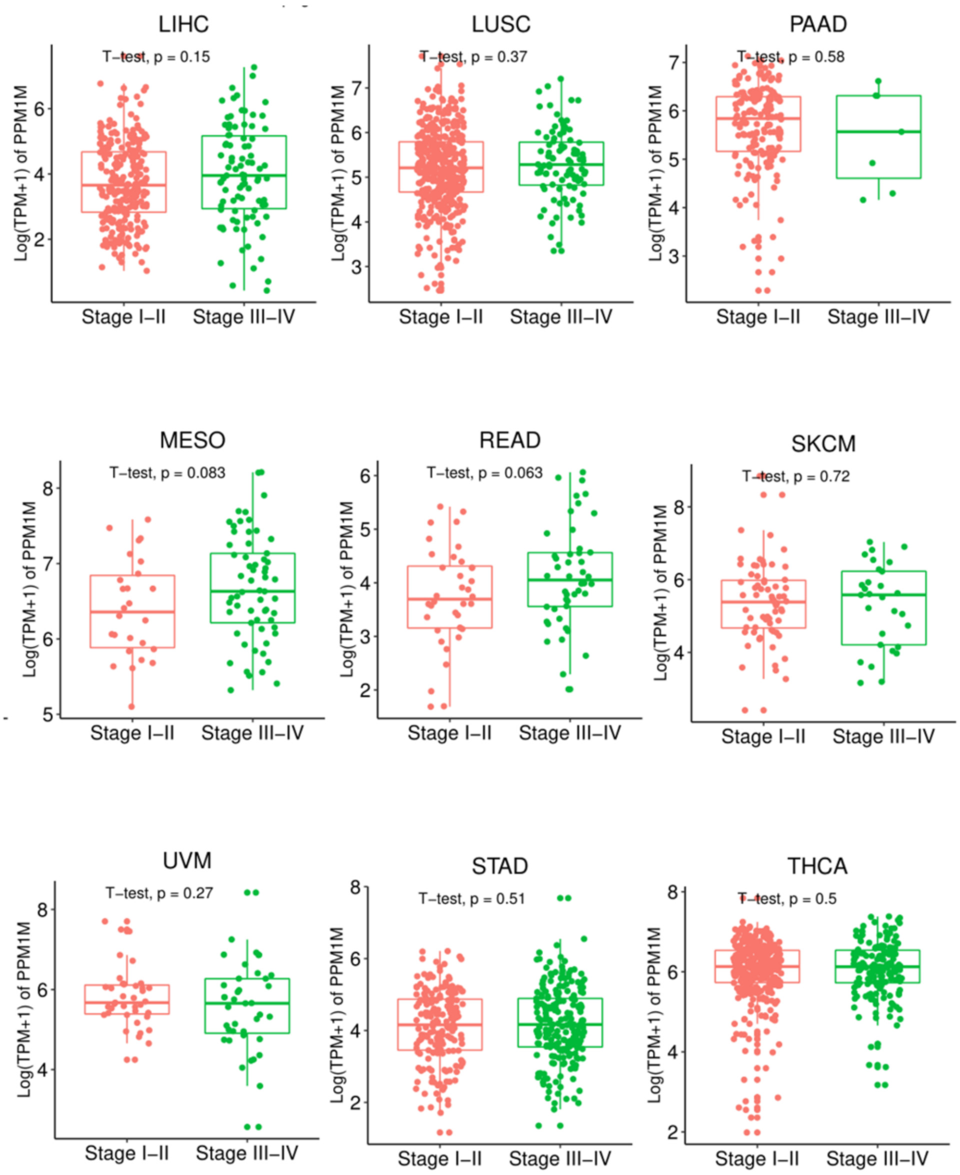

Supplement: Supplementary file 2 [file medi-102-e32758-s002.pdf]
