# Supplementary material for: Exploring the immunological role and prognostic potential of PPM1M in pan-cancer
Source: Medicine (Baltimore). 2023 Mar 24;102(12):e32758. doi: 10.1097/MD.0000000000032758 (PMC10036021; doi:10.1097/MD.0000000000032758)
Supplement: Supplementary file 3 [file medi-102-e32758-s003.pdf]

Supplementary Figure 2: The results of correlation analysis of *PPM1M* expression with the estimated immune scores, stromal scores and estimated scores. (A) Estimated immune scores, (B) Stromal scores, (C) Estimated

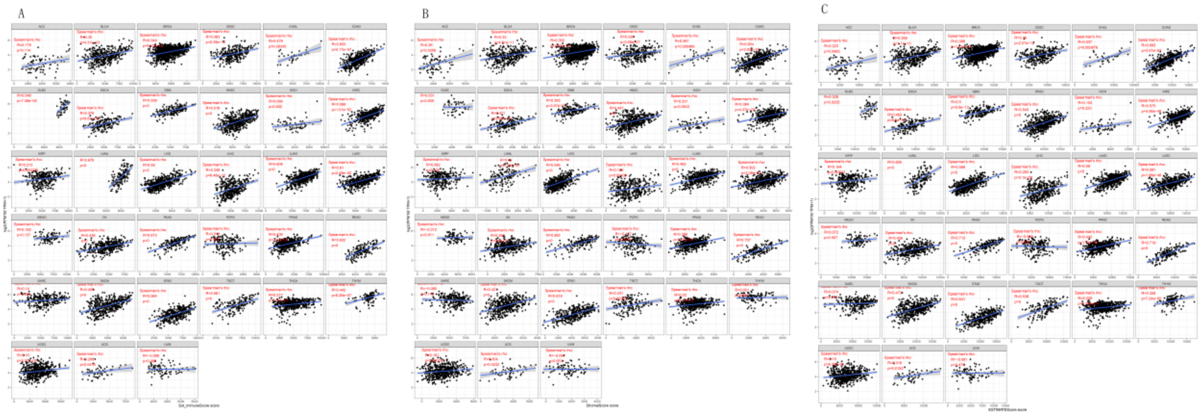

Estimated immune Score   Stromal Score   Estimated Score
